# Vaccines and autism: a preliminary qualitative study on the beliefs of concerned mothers in Italy

**DOI:** 10.1080/17482631.2020.1754086

**Published:** 2020-04-16

**Authors:** Monica Pivetti, Giannino Melotti, Claudia Mancini

**Affiliations:** a Department of Human and Social Sciences, University of Bergamo, Bergamo, Italy; b Department of Education Studies «Giovanni Maria Bertin»(E.D.U.), University of Bologna, Bologna, Italy; c Department of Psychological, Health and Territorial Sciences (Di.S.P.U.Ter.), University of Chieti-Pescara, Chieti, Italy

**Keywords:** Vaccines, autism, beliefs, hesitancy, mothers, content analysis

## Abstract

**Purpose**: While a large body of evidence has shown that the administration of the measles-mumps-rubella (MMR) vaccine is not associated with an increased risk of autism spectrum disorder (ASD), a hesitant attitude towards childhood vaccination is still present among the public. In this study, we aim to investigate the mothers’ perceptions of the cause of their child’s ASD in order to increase our understanding of vaccine hesitancy.

**Methods**: This study draws on the analysis of 18 semi-structured interviews of mothers of children with ASD on the causes of autism.

**Results**: The interview material was content-analysed. The main themes were 1) childhood vaccines; 2) genetics; 3) specific conditions of the mother or the newborn at the moment of delivery; 4) environmental factors such as the mother’s lifestyle or her diet. The link between vaccines and autism was prevalent. About one third of the mothers reported that their child’s ASD was a consequence of a combination of two or more factors, i.e., childhood vaccines and specific conditions of the newborn or the mother at the moment of delivery.

**Conclusion**: This study provides preliminary insights into recurring sets of beliefs concerning the causes of ASD among the mothers of affected children.

According to the Diagnostic and statistical manual of mental disorders (DSM-5®), Autism Spectrum Disorders (ASDs) is a neurodevelopmental disorder characterized by persistent deficits in social communication and social interaction (e.g., deficits in social-emotional reciprocity, nonverbal communicative behaviours, and developing, maintaining, and understanding relationships) that causes clinically significant impairment in social, occupational, or other important areas (American Psychiatric Association, 2013). Furthermore, restricted, repetitive patterns of behaviour, interests, or activities (e.g., simple motor stereotypies, echolalia, rigid thinking patterns) are generally present in ASD. Symptoms must be present in the early developmental period (but may not become fully manifest until social demands exceed limited capacities or may be masked by strategies learned in later life) and they are not better explained by intellectual disability or global developmental delays (DSM-V). Autism is known as a “spectrum” disorder because there is wide variation in the type and severity of the symptoms people experience. ASD occurs in all ethnic, racial, and economic groups. Although ASD can be a lifelong disorder, treatments and services can improve a person’s symptoms and ability to function. According to the Autism Spectrum Disorders in the European Union programme (ASDEU, 2020), the overall prevalence of ASDs’ in Italy is approximately 1:100, in line with other European countries.

The onset of behavioural signs of ASD is usually conceptualized as occurring in one of two ways: an early onset pattern, in which children demonstrate delays and deviances in social and communication development early in life, and a regressive pattern, in which children develop largely as expected for some period and then experience a substantial decline in or loss of previously developed skills. While it was long believed that the majority of children with ASD demonstrated an early onset pattern, more recent studies suggest that regressive onset occurs more frequently than previously recognized, thanks to developments in more sophisticated methodology and research designs (Ozonoff & Iosif, 2019; Thompson et al., 2019). Recent studies have also noted that regression in children with ASD might be under-reported (e.g., Boterberg et al., 2019).

Although the aetiologies for ASDs are unclear, genetics and environment have been identified as contributing factors (e.g., Benvenuto et al., 2009; National Institute of Mental Health, 2019). The beliefs concerning the causes of ASDs among parents of affected children are especially important to understand, as these beliefs may affect the parent-child interaction and parenting, how parents communicate with health care providers and parents’ decisions regarding treatment practices, vaccination practices and future health care (e.g., Elder, 1994; Mercer et al., 2006; Mire et al., 2017). For instance, parents who believed that vaccines contributed to ASD discontinued or changed vaccination practices (Bazzano et al., 2012).

## The origin of the beliefs on the link between vaccinations and ASD and the current anti-vaccination movement

The history of vaccine opposition is a lengthy one, with Cotton Mather and other advocates of variolation in eighteenth-century New England forced to defend immunization practices against the perception that vaccines cause more harm than the diseases that they are meant to prevent. The same applies to Edward Jenner’s smallpox vaccine (Schwartz, 2012).

Over the past few years there has been a great deal of concern regarding the potential links between childhood vaccinations and the development of ASD (Fischbach et al., 2016; Mendel-Van Alstyne et al., 2018; Yaqub, Castle-Clarke, Sevdalis & Chataway, 2014). The vaccinations that have been the focus of most attention are measles-mumps-rubella (MMR) vaccines and thimerosal-containing vaccines such as the diphtheria, tetanus, pertussis (DPT or DT) vaccine. A pivotal role has been played by the publication of Wakefield’s 1998 study in the *The Lancet* claiming that there is a link between the administration of the polyvalent MMR vaccine and the appearance of autism and bowel disease. Subsequently, the study was fully discredited and *The Lancet* retracted the article in 2010, pointing out that elements of the manuscript had been falsified, leading to Wakefield being discredited as a researcher and struck off the medical register (Deer, 2011; Poland & Spier, 2010). Despite the retraction, Wakefield was the main proponent of the movement that started pointing to the MMR vaccines as cause of autism and remains a major influencer in the anti-vaccine movement (Smith, 2017).

As a response to this belief, a number of large-sample rigorous studies has produced a substantial body of evidence showing that the administration of the MMR vaccine was not associated with an increased risk of ASD (Goin-Kochel et al., 2016; Hviid, Hansen, Frish & Melbye, 2019; Jain et al., 2015; Taylor et al., 2014; Uno, Uchiyama, Kurosawa, Aleksic & Ozaki, 2015). Notwithstanding those studies, the beliefs on the link between vaccines and autism has spread to many different parts of the world, especially Western Europe and North America (Dubé et al., 2015; Plotkin et al., 2009). The Internet has become an important source of health information for the public, and it offered an unprecedented opportunity for antivaccination activists to spread their messages to a wider audience and recruit new members (Hobson‐West, 2007; Kitta, 2012). Social media may have a role in spreading anti-vaccination ideas and making the movement durable on a global scale (Smith & Graham, 2019). For instance, individuals who are opposed to vaccination are very active in news forums, resulting in a minority of users generating a disproportionate amount of anti-vaccination content (Pereira et al., 2013).

As a result of the spread and increased acceptance of these arguments, researchers have documented reduced trust in medical practitioners by parents and an increase in concerns about vaccines. The idea that the vaccines are harmful has contributed to a climate of mistrust vis-à-vis all vaccines, to a decline in vaccination rates in the USA and many European countries and the re-emergence of other previously controlled diseases (e.g., Brown et al., 2010; Dardennes et al., 2011; Hussain et al., 2018; Smith et al., 2011).

## Vaccine hesitancy

In recent years, vaccine hesitancy has been defined as a delay in acceptance or refusal of vaccination despite availability of vaccination services. Vaccine hesitancy is complex and context specific, varying across time, place and vaccines. A hesitant attitude towards childhood vaccination means that some parents are doubtful about the benefits of vaccines, worry over their safety and question the need for them. An attitude of hesitancy differs from an action of vaccine refusal. This means that hesitant attitudes are not only limited to those who refuse vaccinations or those who encourage others to refuse vaccinations. Even those who are vaccinated can harbour hesitancy towards certain aspects of vaccination (Enkel et al., 2018; MacDonald, 2015; Peretti-Watel et al., 2014; Wang, Baras, Buttenheim, 2015). Yaqub et al. (2014) found that the most commonly cited reason for hesitancy towards vaccination was safety concerns. Also, a lack of awareness, low perceived severity of illness and a belief in alternative medicine were often cited as reasons for hesitancy.

According to Dube, Vivion and MacDonald (2015), one of the main determinants of parents’ vaccination decisions are their knowledge and attitudes, such as their knowledge and awareness of immunization, the perceptions of the safety/efficacy of vaccines (fear of adverse events), the perception of the risk of vaccine-preventable disease (VPD), their beliefs about immunity (preference for “natural” immunity; “too many, too soon” and immune system overload), perceptions of the importance of vaccination for child’s health (e.g., preference for “natural health”), and anticipated regret (e.g., anticipating feeling of guilt if the child contracts a VPD or suffers from an adverse event).

Vaccine rejection was found to be related to parental beliefs in complementary and alternative medicine (CAM) (Attwell et al., 2018). Reifying “the natural”, these parents eschewed vaccines as toxic and adulterating, and embraced CAM as a protective strategy for immune systems before, during and after illness. CAM emerged as part of an expert system countering Western medicine (Brunson, 2013). Parents viewed their children’s bodies as being naturally perfect and in need of protection, and saw vaccines as an artificial intervention that enters the body unnaturally, via injection. Parents perceived immunity occurring from illness to be natural and superior, while immunity derived from vaccines as inferior and potentially dangerous (Reich, 2016).

Among the general public, the degree of belief in the vaccine–autism linkage was found as the major factor associated with a delay or omission of one or more vaccines among those families (Rosenberg et al., 2013).

As for parents of children with ASD, studies have shown that some of them continue to attribute their child’s autism to immunizations (Chaidez et al., 2018; Fischbach et al., 2016; Hebert & Koulouglioti, 2010; Tomeny et al., 2017). Parents’ beliefs about the causes of ASD varied in terms of the type of onset: congenital versus regressive. Parents more often advocated genetics as the cause for autism when their children exhibited the congenital type, while they advocated external mechanisms (e.g., vaccinations) when their children presented with the regressive type (Goin-Kochel et al., 2015; Goin-Kochel & Myers, 2005).

Vaccines were mentioned as possible causes of autism also among parents from non-Western countries. Alqahtani (2012) found that parents of affected children mentioned that the vaccines could cause autism in Saudi Arabia. Sarrett (2015) found that vaccine-related explanatory models for autism were used by Kerala parents, India. Wolff and Madlon-Kay (2014) reported that Somali parents living in the USA were more likely than non-Somali parents to have refused the MMR vaccine for their child, because they had heard of adverse effects associated with the vaccine.

In our view, it is important to understand the complex role of health beliefs in everyday life, in order to fully address the complexities of health as a cultural and psychological phenomenon and to take into consideration the cultural, social, economic and political determinants of health (Herzlich, 1973; Jovchelovitch & Gervais, 1999). For instance, during the last 15 years, Italy has shown a decrease in vaccine coverage similar to other European countries and it could be an appropriate environment in which to study the health beliefs of parents concerning childhood vaccination.

## The Italian case

Italy is a country with a long-standing tradition of high coverage with compulsory vaccinations. In 2007, some regions (e.g., Veneto) decided to change mandatory immunization to recommended-only immunizations, hoping to maintain high coverage by a spontaneous uptake (Bonanni, 2018). Ten years later, infant immunization coverage decreased, falling below 95% coverage in 2016 (the target set by the Health Ministry). Currently, MMR coverage rates average 87% at the national level, with heterogeneous regional patterns (Signorelli et al., 2017).

Italian adolescents’ perception of the usefulness of vaccines is remarkably low (Pelullo & Di Giuseppe, 2018). Research literature shows that the reasons of this hesitancy include: (a) the influence of the anti-vaccination movements, spreading doubts as to the benefit/risk profile of vaccinations (Burioni, 2016; Innocenzi, 2017); (b) the “balanced” media coverage, giving the same credit to the medical community bringing solid evidence supporting vaccinations and to individuals opposing vaccinations based on claims of serious side-effects (Odone et al., 2015; Odone & Signorelli, 2017); (c) the role played by social media in sharing personal opinions and autobiographical stories involving vaccinated children (Ferro et al., 2015).

In addition to this, three relevant news items received remarkable newspaper and social media coverage: (a) in April 2012, the Court of Justice of Rimini issued a vaccine-injury compensation order establishing a possible link between MMR and autism; (b) in March 2014, public prosecutor in Trani (Bari) started an investigation to establish a causal link between MMR vaccine and autism; (c) in November 2014, a Milan court granted compensation to a boy diagnosed with autism, allegedly caused by hexavalent vaccination.^1^ Also, some studies were published in the 1990s attributing autoimmune diseases to various vaccines (e.g., Cohen & Shoenfeld, 1996; Singh et al., 1993). Those studies relied on samples of limited size, and their results were not replicated in any recent extended epidemiological study.

The Internet and social networks have had a clearly plausible and likely role in the spreading of anti-vaccination attitudes. Aquino et al. (2017), through the analysis of Google Trends, Twitter and Facebook data, showed that 2012 was the tipping point in the public’s confidence in vaccinations in Italy. The highest annual increase in Internet search query data and tweets on vaccines and autism was recorded in 2012. Furthermore, the maximum number of wall posts on anti-vaccination pages and groups, was detected in 2012. Analysing relevant news reported by mainstream Italian media, the decision of the Court of Justice of Rimini in March 2012 was the likely trigger event that led to a spread of vaccine hesitancy in the country. The sentence of the Court of Justice of Rimini was overruled by the appeal at the Court of Bologna in 2015, but this was not given much media publicity.

Even if a large body of literature has ruled out any link between childhood vaccines and autism, is this belief still present in mothers’ representations? Parents are concerned about their children’s health and routinely make health choices for their children (Poltorak et al., 2005). They need to make up their minds about childhood vaccinations. They build their beliefs by being immersed in a social environment made up of contrasting voices coming from general practitioners, social media, the voices of antivaccination movements, court sentences legitimizing the causal role of the MMR vaccine in developing ASD in children, and other parents’ personal accounts (Eskola et al., 2015; Moscovici, 1984). Representations of vaccines as being unsafe and ineffective, as well as negative beliefs regarding the health care system, which was perceived to be untrustworthy, were found by Brown et al. (2010) in their systematic review of the factors underlying parental decisions concerning vaccinations. In Romania, for example, mothers tended to decline HPV vaccinations for their daughters based on the belief that the vaccine represents an experiment that uses their daughters as guinea pigs and the belief that the vaccine embodies a conspiracy theory that seeks to reduce the world population (Craciun & Baban, 2012). Suspicion and conspiracy were found in the central core of parents’ social representations of vaccination in Romania. Parents experienced fear surrounding the hypothesis that vaccines might be part of a conspiracy to decrease world population in order to re-establish the equilibrium between the population and available world resources (Arhiri, 2014). Consequently, it is critical to examine current maternal belief systems regarding the causes of autism.

## Methods

This study draws on the analysis of semi-structured interviews of mothers of children with ASD concerning the causes of autism. The use of the semi-structured interview contributes to the understanding of the life-worlds of respondents and allows for the analysis of “naïve theories” or individual cognitions of participants as expressed through the medium of words. These verbalizations are the means by which the researcher can collect feelings, understandings and explanations of people as they express them (Berg et al., 2004; Gaskell et al., 2000).

### Participants

The sample included 18 mothers of children with an ASD diagnosis, mean age 43 (range 35–48). Ten participants (55.5%) had a high school degree and six (33.3%) a university degree. The majority of participants was married. Sixteen children were male (88.8%) and two were female, from 4 to 17 years, with all meeting the ASD diagnosis criteria (see Table I). Parents did not belong to any parents’ association. We chose not to get in touch with any such parents’ association so as to avoid biasing the sample on the beliefs shared by members of a single association.

**Table I.** The sample

Participant	Gender	Age	Education	Marital status	Sex of the child	Child’s age at the diagnosis(and at the moment of data collection in parenthesis)
1	Female	38	Graduate	Married	Male	2.5 years old (4)
2	Female	48	Upper Secondary school	Married	Male	1.5 years old (4)
3	Female	39	Upper Secondary school	Married	Male	3 years old (5)
4	Female	46	Lower Secondary school	Married	Male	3 years old (14)
5	Female	41	Lower Secondary school	Married	Male	2.5 years old (5)
6	Female	47	Upper Secondary school	Married	Male	3 years old (15)
7	Female	47	Upper Secondary school	Married	Male	2.5 years old (15)
8	Female	36	Upper Secondary school	Married	Male	2 years old (6)
9	Female	44	Graduate	Married	Male	2 years old (11)
10	Female	39	Graduate	Married	Male	3 years old (5)
11	Female	42	Upper Secondary school	Married	Female	2.5 years old (5)
12	Female	43	Graduate	Divorced	Male	3 years old (6)
13	Female	44	Upper Secondary school	Married	Male	5 years old (10)
14	Female	47	Graduate	Married	Female	2 years old (12)
15	Female	48	Upper Secondary school	Married	Male	2.5 years old (9)
16	Female	47	Graduate	Married	Male	3 years old (17)
17	Female	43	Upper Secondary school	Married	Male	3.5 years old (12)
18	Female	35	Upper Secondary school	Married	Male	2 years old (4)

### Procedures

Participants were recruited via a purposive sampling technique, also called judgement sampling, that is the deliberate choice of a participant due to the qualities the participant possesses (Etikan et al., 2016). This involves identification and selection of individuals or groups of individuals who are proficient and well-informed with a phenomenon of interest (Cresswell & Plano Clark, 2011). In addition to knowledge and experience, Bernard (2002) noted the importance of availability and willingness to participate, and the ability to communicate experiences and opinions in an articulate, expressive, and reflective manner. The mothers of affected children were well informed and interested about ASD. As this population is difficult to get into contact with, recruitment from specialized clinics occurs frequently in social science research (e.g., Andersson et al., 2012; Goin-Kochel et al., 2016).

Participants were contacted in various ways: via two specialized therapeutic clinics, via snowballing, via the research assistants’ social networks. As for snowballing, this refers to a non-probability sampling technique in which a researcher begins with a small population of known individuals and expands the sample by asking those initial participants to identify others who could participate in the study. It is particularly used by social scientists who wish to work with a population that is difficult to locate (Noy, 2008; Rockliffe et al., 2018). One mother (participant #18) attending a therapeutic clinic specialized in ASD treatment in Pescara (Abruzzo region), provided the contact information of a further eleven mothers attending the same clinic, who might be willing to take part. In agreement with the Local Health Care Service in Pescara, the clinic provides children with ASD with cognitive behavioural therapy, psychomotricity and speech therapy. The eleven mothers were contacted by phone by a research assistant and asked for their availability to take part. All of them agreed. A research assistant/interviewer scheduled a meeting with each mother a few days later. For each interview, the interviewer asked participants if they knew of anyone eligible. One mother, not attending the clinic, was contacted in this way. Moreover, four mothers were recruited via the research assistant’s social network and one mother was recruited at another therapeutic clinic specialized in ASD treatment. Data were collected between April and June 2015.^2^ Interviews were conducted in Italian. They were mostly face-to-face and only three were run via a phone call (participants #15, 16, 17). The interviews lasted approximately 30 minutes each. Interviews were run in convenient places at the parents’ choice such as their home, the participant’s private office, quiet parks. On the whole, the interviewer (third Author) was welcomed, and the respondents willingly talked about their perceptions and views. During the interviews, the interviewer tried to be sensitive to the language and concepts used by the interviewees and tried to keep the agenda flexible. The interviewer could diverge from the interview guide in order to pursue an idea in more detail and he/she could introduce further questions in order to probe the interviewee’s meanings. After the interviews, the mothers were debriefed, thanked for their participation and dismissed.

Eighteen interviews saturated the representational field and no more new ideas came up in the discussions (Creswell, 1998; Guest et al., 2006; Krueger & Casey, 2000). Content validity requirements were met in that the study group was large enough so that little new material was forthcoming towards the end of the data collection.

Written informed consent was obtained after outlining the study purposes and procedures, indicating the reason for recording the interviews, and assuring the confidentiality of all information provided. The research method complied with the norms of the Code of Ethics of the World Medical Association (Declaration of Helsinki) and the Code of Ethics of the Italian Psychology Association^3^ (Associazione Italiana di Psicologia (A.I.P.), 2018).

### Interview guide

The interview guide was specifically developed for this study, based on previous research on the issue (see Table II) (e.g., Dardennes et al., 2011; Hilton et al., 2007; Smith et al., 2011; Rosenberg et al., 2013). The first part of the interview guide aimed to present the research theme and establish a good connection with the participants. It included questions regarding the child, his/her age, diagnosis, education and daily routine. The second part included questions regarding the mothers’ beliefs on the causes of their child’s autism. The questions regarding mothers’ beliefs were framed according to a bottom-up strategy, aiming to trigger participants’ own points of view, and avoiding to specifically ask for their opinion on the link between vaccines and autism (see Table II). The questions about mothers’ beliefs stated: “*In your opinion, which are the possible causes of the ASD? What could have provoked ASD in your child? Have you ever thought about that? Have you ever spoken with your partner about that? What do your family members say about that? What do other parents of ASD children say about that?*”. The third part pointed to the mothers’ expectations about the child’s future life. Finally, the interviewer posed some socio-demographic questions to the participants (e.g., age, education, marital status, occupation). Data presented herein are focused exclusively on mothers’ beliefs about the causes of ASD.

**Table II.** Interview guide

First part—warm-up questions	How old is your child? What’s his/her name? How is he/she now? Does he/she attend the daycare/school? Does he/she interact with other children? In what way?
Second part—the diagnosis, daily routine and the causes of ASD	When did your child get diagnosed? Did your child have a specific diagnosis? Which diagnosis? Have you noticed any changes in your child’s behaviour before the diagnosis? How did you notice that something “went wrong”? How did you find out? How do you feel about the ASD? Did your daily life change? In what way? Is it difficult or not? In what way? In your opinion, which are the possible causes of the ASD? What could have provoked ASD in your child? Have you ever thought about that? Have you ever spoken with your partner about that? What do your family members say about that? What do other parents of ASD children say about that?
Third part—Conclusion	How do you imagine your child’s future? How do you see your family life in the future? Do you think there will be any improvements in your child or in your family life?
Socio-demographic questions	How old are you now? What’s your level of education? Are you married? Do you have a job? If so, what do you do?

### Analysis of the material

The verbal interactions were audio-taped and transcribed verbatim in Italian, which resulted in approximately 60 pages of single-spaced text. The interview material was content-analysed according to the procedures outlined by Dey (2003) and Flick (2018). Recurring beliefs or explanations represented text units, whether a single phrase or a set of statements. The choice of themes/dimensions followed either a top-down, deductive strategy, with some themes emerging from the literature (e.g., Zuckerman, Lindly & Sinche, 2016), or a bottom-up, inductive approach, with some themes emerging from the data, following repeated reading of the interview transcripts (e.g., Pivetti et al., 2016). For instance, the top-down strategy provided the first two themes, that is 1) childhood vaccines; 2) genetics, whereas the bottom-up strategy provided the third and fourth theme; 3) specific conditions of the mother or the newborn at the moment of delivery; and 4) environmental factors such as the mother’s lifestyle or her diet. Firstly, one research assistant (third Author) and the lead researcher (first Author) experienced in qualitative content analysis went through the first five interview transcripts and generated initial categories independently. The research assistant conducted all the interviews and was familiar with the data corpus. Secondly, the two judges met and compared their coding schemes, discussing their rationale in classifying particular text units within specific themes as well as the appropriateness of the theme labels. Thirdly, the two judges together coded the entire corpus of data according to the initial categories (Miles et al., 2014).

### Ensuring trustworthiness

#### Role of the interviewers and researchers

In this study, one trained research assistant (third Author) was in charge of collecting the data. The research assistant was completing her postgraduate programme in Psychology at a medium-sized university in Italy. The data collection and analysis were part of her final thesis. The research assistant was trained in the interviewing technique by the lead researcher (first Author).

Both research assistant and lead researcher are Caucasian women as are the participants, and have a degree in psychology. The lead researcher has had experience in teaching a course on research methods in social psychology at the postgraduate level. The lead researcher has already conducted and published a number of qualitative studies. They both are familiar with relevant literature on the aetiology of ASD and on parents’ beliefs about the link between vaccine and ASD. The research assistant lives in the same area as the participants.

The second Author is a male Associate professor, enrolled in a different university from the one which the first and third Author belong to. He has teaching experience in qualitative research methods (e.g., interviews) and is experienced in qualitative data analysis. The second Author has already published a number of qualitative studies over the last 10 years, occasionally co-authoring the first Author.

#### Translation issues

The interviews were conducted in Italian. Excerpts of the interview had to be reported in English in the Results section. The primary translation issue in this study was how to express the participants’ meaning in English so the voices of the participants could be heard accurately. To address these issues, the first Author translated all quotations which are listed in the Results section into English and then provided both the English translation and the original Italian quotation to a native speaker English proof-reader. The proofreader checked the accuracy of the translations. The lead researcher spent three years abroad during her PhD and English was her main communication language during that time. She is fluent in spoken English.

#### Ethical conduct

During each step of the research process, care was taken to protect the participants’ confidentiality and shield them from harm relating to issues of respect and dignity. During the interviews, the interviewer showed interest in what the respondent said and encouragement in the form of eye-contact and nodding (Stewart & Shamdasani, 2014).

#### Coding bias

Having more than one person involved with data analysis in a qualitative study helps diminish the effects of researcher bias and thereby supports the credibility and trustworthiness of findings. After the two judges (i.e., first and third Author) coded the entire corpus of data, the lead researcher involved another experienced researcher (second Author) in the data analysis. The two researchers discussed the results until all discrepancies were resolved by consensus. According to the review by Raskind et al. (2019), peer debriefing, that is external review of findings by a person familiar with the study topic, is a common standard of rigour used to explicitly discuss trustworthiness in qualitative articles. Broadly speaking, peer debriefing (or review) is a process by which researchers invite an independent third-party researcher to critically analyse the step-by-step processes and decision-making throughout the study, thereby validating the conclusion drawn (Johnson et al., 2020). Along these lines, the overall data analysis process as well as the main themes were discussed with a fourth senior researcher experienced in qualitative research and data analysis. Her insightful advice is mentioned in the Acknowledgements section.

#### Transferability of the results

To ensure transferability, we have provided detailed information about the context, the participants, data collection and data analysis to guide other scholars in replicating the study (Peterson, 2019).

## Results

### The themes

The analysis of verbal material showed that mothers had deeply and at length reasoned with their partners and family members and mused over the factors influencing their child’s disability and had come up with an articulated set of beliefs about the causes of their child’s autism. The belief in the link between vaccines and autism was still present. The main causes/themes were 1) childhood vaccines; 2) genetics; 3) specific conditions of the mother or the newborn at the moment of delivery; and 4) environmental factors such as the mother’s lifestyle or her diet.

According to Table III, the majority of the interviewed mothers pointed to childhood vaccines as causes of their child’s ASD. Genetics emerged as the second most frequent theme, even if genetics alone was called into question in only a few cases. About one third of the mothers reported that their child’s ASD was a consequence of a combination of two or more factors, i.e., childhood vaccines and specific conditions of the newborn or the mother at the moment of the delivery, or genetics and vaccines. It is also worth mentioning that one third of the participants did not point to vaccines at all, while calling into question the role of environmental factors or genetics, and/or other conditions of the mother/newborn at delivery.

**Table III.** Distribution of the participants across the themes

Participant	Childhood vaccines	Other factors related to the mother or the newborn at delivery	Genetics	Environmental factors
#1				X
#2	X			
#3	X	X		
#4	X			
#5			X	
#6		X	X	
#7				X
#8	X			
#9	X	X		
#10	X	X		
#11	X		X	
#12		X		
#13	X			
#14	X		X	
#15	X		X	
#16			X	
#17	X			
#18	X			

For the sake of clarity, the results are divided into (1) childhood vaccines alone, (2) childhood vaccines, regression in the social and cognitive development of the child and other mother/child conditions, (3) vaccines and genetics, (4) environmental factors: the mother’s lifestyle and/or nutrition. When two themes were interconnected, participants referred in the same text unit to vaccines and other conditions related to the mother/child, or to vaccines and genetics. The same text unit was coded into two or three themes when appropriate.

### Childhood vaccines alone

In our sample, childhood vaccines were indicated by the majority of respondents as being the possible cause of autism. Specifically, six mothers referred to vaccines alone. For instance, participant #4 said that: “*I have wondered about this many times. Sometimes I think about the vaccine. Actually … unfortunately … I’m almost completely convinced, as it seems that the batch my child’s vaccine was taken from, the trivalent, if I’m not mistaken, that you generally get at about 11/12 months, it seems that many children had problems after that vaccine. We cannot give an explanation [for our child’s autism] as we have no cases of autism in the family […]. The vaccine gives me plenty to think about*”. Participant #17 referred: “*I blame the vaccine he was given. Everybody says that it is not possible but to me it’s that one [the vaccine] because when a child is healthy at birth, it’s weird and odd that he should develop those symptoms. Well, if go deeper, nobody knows, I know, the reason why my son is this way now*”.

### Childhood vaccines, regression in the social/cognitive development of the child and other mother/child conditions

Generally, vaccines were called into question in association with mothers’ reporting a regression in their child’s social and cognitive development. Those mothers reported that their child has developed some social skills such as playing with a little sister or singing after the mother, a skill that the child loses as he/she grows older. Regression was defined as losing previously acquired skills or abilities. All the mothers who reported regression pointed to vaccines as causes of autism, except one (participant #7). Some participants openly criticized the practice of mass vaccination and expressed mistrust in health care professionals. About regression, participant #13 pointed out: *“I’m against those vaccines as they may affect the children. Not [provoking] only the ASD but also cases of diabetes are growing. I have some proof of this because my cousin has done some research and it is clear that it was caused by the vaccine. I’m almost sure that this damned vaccine … the vaccinations continue regardless … they can be useful but they can be dangerous too. I think some substance inside those damned vaccines is to blame. I’m sure, even because the change was clear, everything happened after a bout of flu and we don’t know where it came from. One week before I took him, a normal child, to have his hair cut, and the next time, after the vaccine and after the flu, to get his hair cut was like hell”.*

Other mothers were more aware of social pressures against the anti-vax movement and did not express their views openly but it was possible to grasp their views anyway. For instance, participant #18 commented*: “Let’s do this vaccine. After 4 days, maybe longer, he got temperature. Doctor, I said, is it linked to the vaccine? Well, let’s see. I need to see the child. After the appointment, the doctor said that it was an ear infection. Ok, I said, it’s possible, even if it wasn’t the right period as it was spring, and it is odd that a child should get an ear infection in spring. He had a high temperature, 39 degrees, for days. The doctor prescribed antibiotics. After a few days, the child fell ill again. Doctor, I asked, what’s happened? Is this to do with the vaccine? No, it isn’t. It’s an ear infection again. The doctor prescribed antibiotics again. The child got two ear infections in a row. After the ear infections, the child started to change, to spend time alone, he didn’t play with the little sister anymore, he didn’t interact with other children at the nursery. What’s the cause? To me, it’s clear”.*

Vaccines were reported as causes of autism in association with other specific conditions of the newborn or the mother at the moment of the delivery. Participants did not blame vaccines alone, but called into question other events that may have intervened concurrently with vaccines, that were depicted as too aggressive for a vulnerable 18-month-old child. Participant #10 stated*: “It is caused by the aneurysm as it is an artery, that bursts partially in the brain, it’s a cerebral haemorrhage affecting all the brain areas … quite a large ischaemic area […]. I think together with this, the vaccine could have worsened the situation, in the sense that the vaccine might not be the main cause, but a concurrent cause. He had an operation, and took many drugs. Vaccines are drugs, and they are tough on an impaired immune system”.*

In one case, regression in the child development was mentioned in association with a child’s condition at delivery. Participant #9 said that: *“I have often wondered about it. I got the idea that I had a problem at delivery. I had liver problems during pregnancy. I had to do many intravenous drips. I had high blood pressure. I had to be admitted to hospital. They induced the delivery and I had an urgent C-section as the child was dry. I think that this was the problem. Then, after he was born, he had a normal development, and then he had a regression. Here we go back to the vaccines (…) After the first dose of the vaccine, he had a regression, as he had the typical babbling. I used to sing little song to him and he would follow me. He used to say ‘mam’ and after this dose of vaccine he stopped talking. He stopped gazing into my eyes. The delivery was something, and the vaccine was a concomitant event”.*

### Genetics and vaccines

Some mothers reported that genetics caused ASD. In two cases, genetics was reported as the only determinant and ASD was described in one case as a familiar heritage and in one case as an unfortunate incident. For instance, participant #16 pointed out: *“It is a rare genetic problem. There is no genetic problem in the parents’ families. It happened by chance as far as we have understood. There is a gene match with a particular outcome, a non-normal outcome. It’s not hereditary. It’s random. It happens once and it’s hard to understand”.*

Participants were supported in their beliefs about the role of genetics when they came across a relative of theirs carrying a similar health condition. As participant #5 stated: *“I think it’s a congenital thing. Delving deep into the history of my family and that of my husband … we didn’t know that … we discovered that I had a relative of my mother who is really autistic, 100%”.*

Some participants reported that childhood vaccines have worsened the genetic conditions of their children. For instance, participant #11 said: *“I believe it was the vaccine, but then there is my cousin. She has the same problem, and then it could also be a genetic problem. She is the daughter of my mother’s brother. Her second child is autistic, then it could be a genetic problem, partially due to the vaccines and partially due to genetics, if the child is predisposed [to ASD], and you also vaccinate him, for me it’s not good. If I had another child, I wouldn’t vaccinate him, there are too many bad things inside that vaccine.”* She expressed her intention not to inoculate any other child of their own in the future.

### Environmental factors: the mother’s lifestyle and/or nutrition

As causes of autism, two mothers called into question environmental factors alone, such as lifestyle, environmental pollution and nutrition. In their view, autism was caused by an external poison that entered into the mother’s body, and as a consequence affected the child. During pregnancy, the mother might have eaten or breathed in some chemicals influencing the development of the foetus. Participant #1 commented: *“I don’t know. I had the same lifestyle as I had with my [not autistic] first daughter. Sometimes I wonder if I went somewhere when I was pregnant, maybe I ate something infected, whatever it could have been, I don’t know”.*

## Discussion

This study provides some preliminary understanding of the beliefs in a sample of mothers as concerns the causes of their children’s ASD. We investigated whether the narratives of mothers of affected children continue to incorporate the idea that vaccines are unsafe for their children and therefore contributed to the development of ASD, despite the number of epidemiological studies pointing to the absence of any correlation between vaccines and autism.

The causes of autism are a theme of particular relevance for parents of affected children and their beliefs are pivotal factors underlying the decision to vaccinate their children (e.g., Brown et al., 2010; Brunson, 2013; Dardennes et al., 2011). Our participants, the mothers of affected children, are the most informed and affected people in regard to ASD. Previous studies have shown that parents build shared representations about the child vaccines in order to cope with the decision as to whether or not to vaccinate their children (e.g., Craciun & Baban, 2012).

Moreover, parents of affected children are perceived by other parents as authoritative producers of narratives about the causes of autism, and might contribute to spreading a belief concerning vaccines as being toxic for children’s healthy lives (Downs et al., 2008; Mnookin, 2011; Rodriguez, 2016; Venkatramana, Garg & Kumar, 2015; Wolff & Madlon-Kay, 2014). Parents of children who perceive that their children have been harmed by vaccines are becoming frequent actors in media coverage of vaccine debates. Recently, web sites, blogs, email lists, and related social media have allowed parents to instantly compare their experiences and share theories regarding the causative role of vaccines (Aquino et al., 2017; Kang et al., 2017; Tomeny et al., 2017; Ward, Peretti-Watel, Larsone, Raudef & Verger, 2015).

Some five years after the Wakefield study was retracted, the interviewed mothers still pointed to vaccines as being a possible cause of autism and described mass vaccinations as being dangerous for their children’s bodies, in line with the relevant literature on the subject (Freed et al., 2010; Hebert & Koulouglioti, 2010). To support their claims, mothers reported a regressive onset of the disorders, describing the emergence of autistic symptoms following a period of typical development. Children were described as developing normally up until a certain age, at which point they get the vaccination shot and begin to lose previously acquired skills and fail to progress at their former pace (Goin-Kochel & Myers, 2005). Mothers blamed some unspecified component, toxin or contaminant, of the vaccines, not the virus itself, for bringing about the condition.

One novelty of the study lies in the findings that some mothers developed a structured belief system as to the cause of autism, related to the role of vaccines together with other mother/child conditions or to the role of vaccines together with genetics. According to the interviewed mothers, it was not the vaccine itself, but the interaction between the strength of the vaccine and the weakness of the child that causes the autism. Participants blamed the co-occurrence of vaccines and other factors related to the mother/child and the co-occurrence of vaccines and genetic factors. For instance, some mothers felt that the vaccine might be too strong for children who are already debilitated by a difficult delivery or by non-ASD related health problems. The vaccine contributed to exacerbating a condition of weakness in their child (Chen et al., 2014).

A diagnosis of autism is an event that deeply destabilizes family life. Saying that “Genetic factors could largely contribute to autism liability but have proven more complex than initially anticipated due to interindividual heterogeneity, numerous contributing loci, and multiple genes and gene-environment interactions” (Benvenuto et al., 2009), implies a great amount of unpredictability of disease occurrence. Parents generally wonder why it happened to them and not to another family, and what the “real” cause of their child’s autism was. When trying to make sense of the unpredictable nature of ASD, parents blamed vaccines and their toxic components, that is elements external to their child’s integrity, that had affected the wellbeing of their otherwise healthy kid. This way, they managed to downsize the anxiety created by the unpredictable aetiology of ASD.

A group of mothers recognized the role of genetics. Generally, genetics was called into question when parents were aware that other family members had previously been diagnosed with ASD. This led parents to blame genetics in combination with vaccines. This reasoning is in line with the stance of the anti-vaccination movement, which is particularly prone to narratives of risk and uncertainty. According to those narratives, vaccines were perceived as being unsafe due to the risks of adverse events, resulting in severe or lasting medical consequences (Abeysinghe, 2015; Hilton et al., 2006; Tafuri et al., 2014). Among the mothers pointing to genetics, some mothers reported a belief involving both genetics and vaccines as causing autism. Mothers reported that childhood vaccines had worsened a previous genetic condition in their children.

Many studies have shown that scientists and parents differ significantly in regard to beliefs concerning the likely major cause of autism and priorities for further research. Scientists believe in genetic causes while many parents believe in vaccines as being the cause of autism (Fischbach et al., 2016; Joffe, 2002). The viruses and the medical achievements brought by vaccines are not immediately seen by parents, moving the origin of immunization away from the protection of their children’s health. What parents can clearly see is the co-occurrence of childhood vaccines and the regressive onset of ASD (Frith, 2009). They reported that their children used to behave like any other child at the daycare centre, but after the vaccine they started to change. In this sense, parents pointed to an external element that had affected the wellbeing of their otherwise healthy child. The scientific discourse relating to genetics was merged with the rumours about vaccines causing autism, resulting in a mingling of the two discourses implying the interaction of vaccines and genetics in provoking idiopathic illnesses.

It is worth mentioning that, in a few cases, parents pointed to environmental factors as a possible cause of autism. Again, these are factors external to the mother’s body, such as some food the mother may have eaten or chemical elements the mother might have been exposed to during pregnancy. This is in line with previous studies about parents’ beliefs pointing to environmental factors such as environmental pollution or diet, as causes of autism (Chen et al., 2014; Zuckerman, Lindly & Sinche, 2016). Feelings of guilt were not openly expressed by the mothers during the interviews. However, they seem to have extensively questioned themselves about their possible role in the cause of their child ASD.

Laypeople do not have enough time or resources to consult the original sources of scientific knowledge, whether they are scientific textbooks, journal articles, or medical procedures (Gervais & Jovchelovitch, 1998) but, at the same time, they need to make up their minds about health issues such as vaccinations. In this sense, the social representations theory (SRT) could be of help for understanding the beliefs system as to the causes of autism. SRT studies the ways in which scientific knowledge becomes simplified and popularized in common sense knowledge, given that knowledge based on scientific methods is difficult to understand for the non-specialist (Bangerter, 2000; Howarth, 2006; Jaspers & Fraser, 1984; Joffe, 2002; Moscovici, 1984, 1988). Future research could explore the content and the structure of the social representations of childhood vaccines in general population. The current practice in SRT would suggest studying how the different beliefs are anchored to psychological, psychosocial and/or sociological variables, in line with the research strategy used by Doise et al. (1992; e.g., Pivetti, Melotti & Bonomo, 2017).

As for the implications for policy and practice, the most effective interventions to address vaccine hesitancy were those tailored to a specific population and their specific concerns (Jarrett et al., 2015). Parents point to vaccines and genetics, or vaccines and a mother’s condition as co-occurring determinants of their child ASD. This study advises health professionals to take into consideration the parents’ structured set of beliefs about childhood vaccines, not only a single issue. Healthcare workers should try to understand and be open to parents’ perceptions and feelings rather than quickly dismissing them. Identifying parents’ beliefs about their child’s illness may be an important step in formulating family interventions to facilitate appropriate care, reduce distress and enhance well-being (Dardennes et al., 2011).

As a number of parents believe their child’s condition is genetic, it may also be helpful for providers to explore autistic-like traits among other family members. For instance, providers might ask parents if they have ever noted that other family members have difficulty with social communication or repetitive interests and/or behaviours. Parents may find discussing this topic will help them understand more about why their child has ASD or what their child’s future might be like.

### Study limitations

Among the many limitations of our study, we have to mention the nature and the size of the sample. Small sample size may create problems in qualitative research, given that the smaller the sample size, the more likely it is that the perceptions solicited and gathered will be limited and may bias the results either upward or downward. On the other hand, the larger the sample size, the less chance of failure in terms of failing to uncover perceptions or opinions that researchers might want to know. A larger sample would provide more depth in grasping the wide spectrum of parents’ points of views. For instance, the voices of so-called “anti-vax parents” could be compared to the voices of the parents supporting vaccinations, in order to highlight commonalities and differences.

As concerns the sample composition, we must acknowledge that 12 participants were attending the same clinic. This subsample could share a specific view about the causes of ASD. The results show that mothers believing in the genetic causes of autism and those believing in the vaccine causing autism were included in the data collection. Furthermore, in order to rule out the possibility that the subsample of participants recruited via the same clinic and the subsample of participants recruited via researchers’ network or snowballing might not be comparable, we counted how many participants endorsed vaccines as causes of autism among the subsample of 12 participants recruited from the same clinic and in the subsample of 6 participants recruited via the researcher’s network or snowballing. The percentage of participants endorsing vaccines was the same, that is 66.6%, in the subsample of participants recruited from the clinic (8/12) and in the subsample recruited via researcher’s network and snowballing (4/6). We consider this to be an indication that the two subsamples were comparable and not too unlike each other.

Another study limitation lies in the lack of exploration of the source participants generally used to gather information on ASD. As we mentioned in the Introduction, the Internet and social media play a strong role in the spreading of anti-vaccination attitudes. Moreover, social representations theory (SRT) refers to everyday conversations as a way to build a shared understanding of the new technologies. Future studies should better explore from where and how parents of affected children collect information and build their beliefs about the causes of ASD. Future research into beliefs concerning the causes of autism could also investigate the parents’ vaccination practices, if they are going to delay their child’s future vaccines or if they will forego future vaccines. Finally, on one hand future studies could also explore the relation between the age of diagnosis or age of concerns by parents and, on the other hand, the beliefs of parents concerning the causes of ASD. The temporal continuity between vaccination and symptoms of ASD may have driven parents to blame vaccines as being the cause of their child’s ASD. These data could lead to a better understanding of the belief system concerning the causes of autism, vaccine hesitancy, and their relations with social practices.

## Conclusions

The current study provides preliminary insights into recurring sets of beliefs concerning the causes of ASD among the mothers of affected children. More studies are needed to tailor education initiatives and media campaigns, capable of reconciling the two competing needs: societal needs for herd immunity and the individual perception of vaccines as being dangerous and risky.

## Notes

1. A hexavalent vaccine, or 6-in-1 vaccine, is a combination vaccine with six individual vaccines conjugated into one, intended to protect people from multiple diseases: diphtheria, tetanus, pertussis, hepatitis B, poliomyelitis and Haemophilus influenzae type b (European Medicine Agency, 2010).

2. Data were collected two years before the Italian National Immunization Prevention Plan 2017–19 was introduced, requiring a proof of vaccination when enrolling children in kindergartens and schools. For this reason, we did not mention the current Italian law in the Introduction section.

3. Currently, there is no Local Review Board for Research in Psychology, at the University of Chieti-Pescara.
